# Mental Health Needs of an Emerging Latino Community

**DOI:** 10.1007/s11414-020-09688-3

**Published:** 2020-01-30

**Authors:** Linda Bucay-Harari, Kathleen R. Page, Noa Krawczyk, Yvonne P. Robles, Carlos Castillo-Salgado

**Affiliations:** 1 Department of Epidemiology, Global Public Health Observatory, Johns Hopkins Bloomberg School of Public Health, 615 N. Wolfe Street. Room E6136, Baltimore, MD 21205 USA; 2 Division of Infectious Diseases, Johns Hopkins University School of Medicine, Baltimore, MD USA; 3 Department of Mental Health, Johns Hopkins University and Johns Hopkins Bloomberg School of Public Health, Baltimore, MD USA

**Keywords:** Mental health, Migration, Immigrant community, Latino health, Undocumented immigrant, Social health inequities

## Abstract

Over the last decade, Baltimore has become a non-traditional sanctuary city, receiving an unprecedented influx of Latino immigrants, mostly from Central America’s Northern Triangle, who are often fleeing violence in their home countries. This study explored the nature and frequency of healthcare utilization for mental health problems among uninsured/uninsurable Latinos who received outpatient care between 2012 and 2015 through an academic hospital-affiliated program that covers primary and specialty services to uninsured patients without regard to documentation status. Encounters for mental health disorders were the most common category, accounting for 14.88% of all visits. Mood (78%) and anxiety disorders (16%) were the most prevalent mental health diagnoses. The most frequent reason to seek care was symptom, signs, and ill-defined conditions (37.47%), and within this subgroup, pain was the leading cause of seeking care (88%), which may indicate high rates of somatization of mental health distress. This study presents a unique opportunity to explore the burden and nature of mental health needs among a population for which healthcare information is rarely attainable and highlights the need for culturally competent screening mechanisms and interventions to address the stressors faced by emergent communities.

## Introduction

Latinos are the largest and fastest-growing minority in the USA. In 2015, 17.6% of the population (56.6 million people) were identified as Hispanic or Latino,^1^ and by 2060, this proportion is estimated to increase to 28.6%.^2^ US-born Latinos outnumber foreign-born Latinos in most states with the exception of the District of Columbia and Maryland^3, 4^, and around 11 million Latino immigrants in the USA are undocumented.^5^ Immigration trends have changed in recent years, with an unprecedented number of people coming from Honduras, El Salvador, and Guatemala, a region known as Central America’s Northern Triangle (NT), shifting the social fabric of some communities that are traditionally mostly comprised of individuals of Mexican descent and forming new ones.^6–8^ Latino immigrants have traditionally moved to border states or regions with large well-established Latino communities and strong social support networks for newly arrived populations. In the last two decades, however, the influx of Latino immigrants has expanded to non-traditional areas, such as Baltimore City, where the Latino population increased 135% between 2000 and 2010.^9^ According to the Maryland Department of Health and Mental Hygiene, in 2013 around 40% of the Latino population in Maryland was from the NT.^10^ Immigrant communities in emergent settlement areas like Baltimore face challenges due to a lack of social support and culturally and linguistically competent services that are more accessible in traditional Latino destinations.

Migration is a process that involves several stressors.^11^ Undocumented immigrants are particularly vulnerable since they face unique risks and exposure to traumatic experiences before, during, and after migrating that put them at high risk of mental health distress.^12–15^ The NT countries have among the highest social inequality rates in the Western Hemisphere and homicide rates in the world^6, 16^ In 2012, all three NT countries had among the top 5 highest murder rates in the world,^6^ and hundreds of thousands have fled the increasing violence in the area by migrating north, either to Mexico or the USA. In addition, the journey through Mexico is particularly risky for Central American migrants.^15, 17^ A high proportion suffer unintentional injuries and various forms of violence, such as theft, extortion, mugging, kidnapping, trafficking, and sexual abuse perpetrated by Mexican gangs and federal officials that take advantage of their vulnerable status.^15, 17, 18^ Once in the USA, immigrants face additional stressors related to their documentation status, language and cultural barriers, fear of family separation, discrimination, and limited access to healthcare and public services.^19, 20^

Despite these challenges, some evidence indicates that Latin American immigrants living in the USA have better overall health than US-born Latinos and non-Latino Whites.^21^ A study that analyzed data from a national representative survey found that Mexican immigrants had a lower lifetime risk of developing mental disorders compared to US-born Latinos and non-Latinos.^22^ This phenomenon has been described as the “Hispanic paradox,”^21^ which is often attributed to either a “healthy migrant effect,”^23^ in which migration itself requires a physical and mental level of fitness and is a self-selecting process or that coping skills inherent to Latino culture such as positive reframing and religion may be protective factors against stress and mental health problems.^24^ Some researchers, however, have argued that aggregating Latinos into one group might hinder the identification of specific vulnerabilities and protective factors among subgroups.^11^ It has been reported, for example, that health among Latinos varies based on country of origin, specific mental health disorder, time living in the USA, and other socioeconomic variables.^25^

Assessing mental health among this population is highly relevant in today’s sociopolitical climate, given recent changes to border control, refugee, and immigration policies that may impact the experience of new Latino immigrants to the USA. In addition, older studies do not reflect the impact on the mental health of immigrants related to changes in immigration policy over the last two decades, starting with the Bush administration (2001–2008) deporting 2 million immigrants, continued by the Obama administration deporting 3 million immigrants (2009–2016),^26^ and followed by increasing anti-immigrant rhetoric and the zero-tolerance policy of the Trump Administration.^27^ Migrants, including families with children, facing violence and extortion continue to migrate to the USA in hopes of receiving asylum protection.^28^ However, the Department of Justice has been asked to prosecute “all adult aliens apprehended crossing the border illegally, with no exception for asylum seekers or those with minor children.”^27^ The deleterious impact of these policies on the mental health of immigrants is beginning to be documented in the literature.^29^ For example, recent studies indicate that Latina mothers have reported high rates of anxiety, stress, and depressive symptoms caused by different stressors such as persecution, physical and sexual abuse, extortion, and separation from their children.^30, 31^

There is limited information about healthcare utilization for mental health disorders among undocumented immigrants. This is largely due to formidable barriers to healthcare for this population due to restrictions in access to public health insurance, as well as limited to private and employer-based health insurance.^32^ Uninsured patients have also shown to face poor access to care, quality of care, and health outcomes.^33, 34^ Gaining a better understanding of the mental health needs and the way in which this group reports symptoms of emotional distress will help providers and health systems to better serve the needs of immigrants, especially in communities such as Baltimore that are facing unprecedented new waves of immigration.

In 2009, Johns Hopkins Medicine established the Access Partnership (TAP) to provide specialty services to uninsured residents of the East Baltimore community surrounding Johns Hopkins Hospital and Johns Hopkins Bayview Medical Center.^35^ Since the expansion of Medicaid through the Affordable Care Act, and as uninsurance rates have declined in Maryland,^36^ undocumented Latino immigrants (who are ineligible to enroll in Medicaid) have become the primary utilizers of TAP, providing a unique opportunity to evaluate patterns of healthcare utilization among uninsured/uninsurable Latinos who qualify for the program. To be eligible for the TAP program, patients must be referred to care by a community primary care provider, be uninsured/uninsurable, and demonstrate financial need. The purpose of this study is to explore the nature and frequency of healthcare utilization for mental health and related problems among uninsured/uninsurable Latinos who received outpatient care at the Johns Hopkins Hospital and Johns Hopkins Bayview Medical Center through the TAP program in Baltimore City between 2012 and 2015.

## Methods

Data for TAP-enrolled male and female patients over age 16 from outpatient visits at the Johns Hopkins Hospital and Johns Hopkins Bayview Medical Center were analyzed. An integrated database of demographic and healthcare utilization information for all outpatient encounters among patients who identified as Latino and received care through the TAP program between 2012 and 2015 was used. Descriptive statistics were used to illustrate the demographic characteristics of the patients and their medical diagnoses using the International Classification of Diseases, Ninth Edition (ICD-9)^37^ and non-personal identifiers that allowed the integration of the data. This study was approved by the Johns Hopkins School of Medicine Institutional Review Board.

All ICD-9 categories and codes were specified for each patient encounter in the dataset. First, the number and proportion of encounters were calculated by gender and by broad ICD-9 categories in order to assess the frequency of different types of health conditions for which care was being sought among the population of interest. To analyze the frequency of encounters for specific mental health diagnoses, the ICD-9 mental disorder category was further subdivided into six broad diagnostic categories based on how the codes were used in the *Diagnostic and Statistical Manual of Mental Disorders, Fourth Edition (DSM-IV).*^38^ These versions were used on the present report because the ICD-10 and DSM-V were only used widely after the data collection period. The DSM provides a more comprehensive framework to understand the most important groups of mental health disorders. This reclassification was based on the methods used by Hogue and colleagues in 2002^39^ and include the following eight categories: substance-related disorders, adjustment disorders, mood disorders, personality disorders, psychotic disorders, anxiety disorders, somatoform/dissociative disorders, and other mental disorders.

## Results

Between 2012 and 2015, a total of 1735 patients received outpatient medical care through the TAP program, generating a total of 17,010 encounters (with a median of 5 visits per patient). Of all patients in the TAP database, 870, or 50%, identified as Hispanic/Latino, accounting for 6464 or 38% of the encounters in the database. Among encounters involving Latino patients, the average age was 44 years old (range 16 to 82), and females received a greater proportion of care (57%) than males.

A total of 65 patients, or 7.47% of all Latino patients in the dataset, received a mental health diagnosis (Table 1). However, healthcare utilization (number of visits/encounters) was highest for mental health disorders compared to other diagnostic categories, accounting for 784 or 14.88% of all encounters among Latinos. Encounters for symptoms, signs, and ill-defined conditions (SSIDC) were the second highest diagnostic category (10.82%), followed by a range of other health conditions, presented in order of frequency in Table 1.

**Table 1:** Number of encounters by ICD-9 category, among patients with Hispanic origin 2012–2015

ICD-9 Category	Female encounters	Males encounters	All encounters *N* (%)	Number of unique patients *N* (%)
Mental disorders	525	259	784 (14.88%)	65 (7.47%)
Symptoms, signs, and ill-defined conditions	339	231	570 (10.82%)	326 (37.4%)
Neoplasms	264	299	563 (10.69%)	71(8.16%)
Diseases of the nervous system and sense organs	239	237	476 (9.04%)	174(20.00%)
Encounter for specific procedures and after care	238	185	423 (8.03%)	96(11.03%)
Diseases of the genitourinary system	271	130	401 (7.61%)	187(21.49%)
Endocrine, nutritional, metabolic diseases, and immunity disorders	172	114	286 (5.43%)	103(11.84%)
Diseases of the digestive system	95	139	234 (4.44%)	116(13.33%)
Diseases of the musculoskeletal system and connective tissue	76	129	205 (3.89%)	114(13.10%)
Complications of pregnancy, childbirth, and the puerperium	196	–	196 (3.72%)	60(6.90%)
Diseases of the skin and subcutaneous tissue	99	82	181 (3.44%)	51(5.86%)
Persons w/o reported diagnosis encountered during exam	99	79	178 (3.38%)	146(16.78%)
Diseases of the circulatory system	66	97	163 (3.09%)	58(6.67%)
Injury and poisoning	34	86	120 (2.28%)	72(8.28%)
Encountering related to reproduction and development	97	1	98 (1.86%)	26(2.99%)
Infectious and parasitic diseases	25	42	67 (1.27%)	31(3.56%)
Diseases of the respiratory system	31	24	55 (1.04%)	34(3.91%)
Persons with a condition influencing their health status	26	28	54 (1.03%)	29(3.33%)
Congenital anomalies	20	32	52 (0.99%)	26(2.99%)
Encounter health services in other circumstances	29	18	47 (0.89%)	39(4.48%)
Persons w/hazards related to communicable disease	8	32	40 (0.76%)	23(2.64%)
Missing category	16	13	29 (0.55%)	25(2.87%)
Diseases of the blood and blood-forming organs	26	2	28 (0.53%)	11(1.26%)
Persons w/hazards related to personal and family history	10	3	13 (0.25%)	11(1.26%)
Other suspected conditions not found	3	–	3 (0.06%)	3(0.34%)
Multiple gestation placenta status	1	–	1 (0.02%)	1(0.1%)
Other specified exposures and history presenting health hazard	–	1	1 (0.02%)	1(0.1%)
Total	3005 (57%)	2263 (43%)	5268 (100%)	870 (100%)

Most mental health encounters occurred in a psychology community physician setting (84%), followed by the intensive outpatient community psychiatry program (11%), which is a specialty service with several levels of intensity formed by a team of therapists and psychiatrists. Females accounted for the majority of mental health encounters (67%, Table 2). The majority (78%) of mental health encounters was for mood disorders, with 530 encounters for major depression and depressive disorders. Anxiety disorders accounted 16% of mental health encounters, followed by adjustment, psychotic, and alcohol-related conditions.

**Table 2:** Detailed encounters with ICD-9 codes for mental disorders according to DSM-IV categories, among patients with Hispanic origin 2012–2015

	ICD-9 codes	Females	Males	All encounters *N* (%)
Mood disorders		402	206	608 (77.55%)
Major depression	296.20, 296.22, 296.30, 296.31, 296.32, 296.33296.34, 296.35, 296.36	310	164	474
Depressive disorder (NEC)	311	44	12	56
Bipolar disorder	296.40, 296.45, 296.5, 296.6, 296.7	37	5	42
Other episodic mood disorder	296.80, 296.89, 296.90	11	25	36
Anxiety disorders		84	44	128 (16.33%)
Anxiety, unspecified	300.00	24	16	40
Panic disorders	300.01, 300.21	34	1	35
Post-traumatic stress disorder	309.81	23	10	33
General anxiety disorder	300.02	3	17	20
Adjustment disorders		26	1	27 (3.44%)
Adjustment, unspecified	309.9	9	1	10
Adjustment w/ disturbance	309.24, 309.28	4	0	4
Adjustment w/ depressed mood	309.0	13	0	13
Psychotic disorders and symptoms		7	1	8 (1.02%)
Depressive-type psychosis	298.0	6	0	6
Unspecified psychosis	298.9	1	0	1
Paranoid-type schizophrenia	295.30	0	1	1
Alcohol-related disorders and symptoms		0	5	5 (0.64%)
Alcohol withdrawal	291.81	0	1	1
Non-dependent alcohol abuse	305.00	0	4	4
Other mental disorders and symptoms		6	2	8 (1.02%)
Conduct disorder	312.00	0	1	1
Psychosexual disorders	302.70, 302.75	1	1	2
Tension headache	307.81	1	0	1
Persistent insomnia	307.42	1	0	1
Bulimia nervosa	307.51	1	0	1
Unspecified non-psychotic	300.9	2	0	2

The most frequent reason to seek care among Latino patients was SSIDC (37.47%). Previous research has linked SSIDC with physical non-specified symptomatology that can be rooted in mental health distress.^40, 41^ A high number of isolated signs and symptoms were found, and the ones associated with mental health causes^42–44^ were selected and categorized according to their clinical manifestation. Within this subgroup of categories, pain was the leading cause to seek care (88%). The most common form of reported pain was abdominal (55.91%), followed by headache, head and neck symptoms (19%) and chest (10%). Females also made up the majority of patients with SSIDC (61%).

### Implications for Behavioral Health

This descriptive study found high levels of healthcare utilization for mental health disorders – particularly depressive and anxiety disorders – among uninsured/uninsurable Latino immigrants in an emerging community. It also presents a unique opportunity to explore the burden and nature of health and mental health disorders among an emerging Latino community for which healthcare information is rarely attainable. Most of the available research on Latino mental health has been conducted in regions where there are larger and well-established communities composed mainly by Mexican immigrants, with stronger social networks, and, in some cases, more available health and social services. Latino immigrants living in new settlement areas, especially those without documents and as immigration policy has toughened, face considerable stressors that might place them at risk for mental health disorders. Previous studies have shown that Latinos are less likely to seek and access mental healthcare than other racial/ethnic groups.^14, 45^ We found that only 7.47% of Latinos who accessed the TAP program were referred to mental health services, which is consistent with previous findings that suggest that immigrants (Hispanic and non-Hispanic) have lower lifetime risk of developing psychiatric disorders than natives, pointing out how the “Hispanic” or “immigrant” paradox influences mental health outcomes. Still, mental health visits accounted for the largest number of encounters and utilization of healthcare in the TAP program. This might indicate a higher mental health symptom severity among this group. Alternatively, this may also reflect a lack of effective and culturally appropriate services that may lead to continued symptomology and repeated use of services. Mood and anxiety disorders emerged as the most common mental health problems among this population, which is consistent with general global population-level prevalence estimates in which mood and anxiety disorders are often the most common mental health conditions.^46^

The TAP patient population of uninsured/uninsurable individuals due to documentation and socioeconomic status may be particularly vulnerable to mental health disorders given their unique experiences before and after migrating to the USA. Family separation associated with migration may be particularly stressful for people arriving in emergent immigrant areas with less established social networks and services than more traditional settlement areas. In addition, exposure to violence is common in this population and has shown to cause profound damage in the physical and mental health of the victims,^47^ commonly expressed as anxious and depressive symptomatology.^48, 49^ Moreover, populations that face the cumulative effect of poverty, malnutrition, lack of political representation, and other socioeconomic stressors might suffer an exacerbation of their prior mental health distress when they are exposed to other forms of violence such as armed conflict.^50^ Indeed, previous evidence shows that the new wave of migrants from the NT have significant mental health symptoms as consequence of violence and persecution. A study conducted at the US-Mexico border^48^ showed that the vast majority (83%) reported violence as a reason to migrate and at least half were exposed to highly traumatic experiences in their home countries. Respondents in this study reported high rates of psychological distress (one third PTSD, one quarter major depressive disorder, and 17% reported symptoms that satisfied criteria for both conditions).

Latinos have also been shown to be more likely than non-Latinos to somatize psychological distress ^41, 43^ and tend to rely on the general medical sector rather than seeking specialty mental health services.^51^ This information is important to note given that SSIDC was both the most common diagnostic category to seek care among Latinos receiving TAP services and second most common health service encounter. This ICD-9 category refers to symptoms, signs, and laboratory results where no diagnosis classifiable elsewhere was recorded and could potentially be related to a certain degree with mental distress.

Members of different sociocultural groups have shown to experience stress and emotional suffering in diverse ways.^42, 52^ In some cases, symptoms fit the description of a specific disorder according to the DSM, but at other times, affective distress can be expressed as an undefined and generalized form of anxiety or depression.^43, 53^ Medically unexplained symptoms represent an important burden to the healthcare system, and it has been estimated that they account for approximately a quarter to half of all patient visits in primary care settings in the USA.^41, 43, 54^

In clinical settings, SSIDC such as that found frequently in this study may present as isolated symptoms, but it has been argued that in some cultural contexts, these symptoms might be more sensitive indicators of traumatization than those warranting a psychiatric diagnosis (e.g., PTSD).^42^ Since the 1980s, the concepts of “idioms of distress,” “cultural syndromes,” and “culture bound syndromes” have opened research lines focused on understanding the different ways in which diverse sociocultural groups communicate individual and collective suffering via reference to shared beliefs and local ideas about the functioning of the body and mind.^42, 52, 53, 55^ For example, previous literature has described an idiom of distress common among Latinos, *padecer de los nervios*, which is usually characterized by chronic anxiety, pain, depression, somatization, and/or dissociation, as well as acute symptoms such as palpitations, shortness of breath, or trembling.^44, 56^

Further research is needed to understand the different markers or expressions of how members of emerging communities face adversity and vulnerability, which can inform the development of better screening mechanisms and tools to identify mental health problems in a culturally appropriate way, improving the detection of those in need of services.^52^ As the US population grows more diverse, it is important to develop culturally and linguistically competent services and a diverse healthcare workforce that are familiar with the culturally specific manifestations of mental health distress among different groups, so they can frame the treatment options in an appropriate and non-threatening way.^57^ Also, as suggested by Kaiser and colleagues,^52^ incorporating this knowledge into effective public health communication to target specific populations can decrease stigmatization around psychopathology and promote treatment-seeking behavior. More accurate detection can also increase effective care delivery.^42, 58^

In our study, Latino women were more likely than men to use health services in general and mental healthcare in particular. This is consistent with previous studies that show that men are less likely to seek treatment than women, even when they are experiencing the same levels of distress, a tendency that is even more pronounced among men from racial and ethnic minority backgrounds.^59^ One reason men may underutilize mental health services is that they have less favorable attitudes toward mental health services, rooted in traditional masculine norms and stigma surrounding mental health problems.^60^ Additionally, women may have had a higher utilization of healthcare compared to men because pregnancy is an important point of entry to healthcare services, and many women in Baltimore’s Latino community are of reproductive age.^4^

The present findings should be interpreted in light of several limitations. The TAP database had limited information about the demographic characteristics of the patients, such as country of origin, documentation status, and time living in the USA. However, the eligibility criteria for TAP (uninsured/uninsurable) selects for people without documentation status since the expansion of Medicaid through the Affordable Care Act. Nonetheless, the generalizability of this data is limited to those who have access to TAP, which excludes the most vulnerable and disenfranchised migrants who do not have a primary care provider to refer them to TAP. These migrants may not have the social capital to seek care, and the inability to reach these individuals suggests that our findings may underestimate the burden of disease. The database also lacks information on severity of disorder and type of treatment received, as well as data about the nature, intensity, duration, and effects of treatment. Despite these limitations, this paper presents valuable information on the mental health needs of a unique and growing Latino community in the mid-Atlantic. The presented clinical manifestations in the particular case of an emerging community with a high proportion of immigrants from the NT suggest that these new communities have important mental health needs that need to be further studied. Studying factors, such as country of birth, time living in the USA, and acculturation, might be important in learning more about the extent to which the “Hispanic paradox” plays a role in mental health outcomes among emerging Latino communities comprised of non-Mexican immigrants. Given the increasing number of immigrants arriving from the NT in Baltimore and other cities in the USA,^61^ the medical and public health community must be aware and receptive to the type of unmet mental health needs of this marginalized population.

Finally, it is important to acknowledge that, as emphasized by multiple authors, Latinos are not a monolithic group.^11^ The history of each Latin American country entails complex historical, cultural, and social dynamics. Therefore, the study of contextual factors among different Latino groups, such as prior immigration experiences, exposure to violence during migration, as well as the development of social support networks and access to services during resettlement, will allow us to design comprehensive public health programs to address mental health needs of communities across the USA.
